# The need of radiotherapy optimization for glioblastomas considering immune responses

**DOI:** 10.1007/s11604-023-01434-x

**Published:** 2023-04-18

**Authors:** Kentaro Nishioka, Shuhei Takahashi, Takashi Mori, Yusuke Uchinami, Shigeru Yamaguchi, Manabu Kinoshita, Masaaki Yamashina, Hajime Higaki, Katsuya Maebayashi, Hidefumi Aoyama

**Affiliations:** 1 Department of Radiation Oncology, Hokkaido University Hospital, North-15, West-7, Kita-ku, Sapporo, Hokkaido 060-8638 Japan; 2 Department of Neurosurgery, Hokkaido University Hospital, Sapporo, Hokkaido Japan; 3 Department of Neurosurgery, Asahikawa Medical University, Asahikawa, Hokkaido Japan; 4 Department of Radiology, Asahikawa Medical University, Asahikawa, Hokkaido Japan; 5 Division of Radiation Oncology, Nippon Medical School Hospital, Tokyo, Japan

**Keywords:** Glioblastoma, Radiotherapy, Target definitions, Immunotherapy, Lymphopenia

## Abstract

Glioblastoma is the most common of malignant primary brain tumors and one of the tumors with the poorest prognosis for which the overall survival rate has not significantly improved despite recent advances in treatment techniques and therapeutic drugs. Since the emergence of immune checkpoint inhibitors, the immune response to tumors has attracted increasing attention. Treatments affecting the immune system have been attempted for various tumors, including glioblastomas, but little has been shown to be effective. It has been found that the reason for this is that glioblastomas have a high ability to evade attacks from the immune system, and that the lymphocyte depletion associated with treatment can reduce its immune function. Currently, research to elucidate the resistance of glioblastomas to the immune system and development of new immunotherapies are being vigorously carried out. Targeting of radiation therapy for glioblastomas varies among guidelines and clinical trials. Based on early reports, target definitions with wide margins are common, but there are also reports that narrowing the margins does not make a significant difference in treatment outcome. It has also been suggested that a large number of lymphocytes in the blood are irradiated by the irradiation treatment to a wide area in a large number of fractionations, which may reduce the immune function, and the blood is being recognized as an organ at risk. Recently, a randomized phase II trial comparing two types of target definition in radiotherapy for glioblastomas was conducted, and it was reported that the overall survival and progression-free survival were significantly better in a small irradiation field group. We review recent findings on the immune response and the immunotherapy to glioblastomas and the novel role of radiotherapy and propose the need to develop an optimal radiotherapy that takes radiation effects on the immune function into account.

## Introduction

Glioblastomas (GBM) are the most common type of malignant primary brain tumor and the so-called Stupp regimen (maximal tumor resection followed by chemo-radiotherapy with temozolomide (TMZ) and conventionally fractionated radiotherapy (60 Gy in 30 fractions)) is the standard treatment. However, even with recent advances in treatment techniques and therapeutic drugs, no significant improvement in patient survival has been obtained, and it is one of the tumors with the poorest prognosis.

Since the advent of immune checkpoint inhibitors (ICI), attention has focused on the immune response of the tumors, and treatments modifying the immune system have been attempted for various tumors including GBM. In addition, it has been reported that lymphopenia during treatment correlates with the prognosis of various cancer types, and that lymphopenia may attenuate the effects of ICI. In high-grade gliomas, including GBM, there is a report that the decrease in CD4-positive lymphocytes during treatment correlates with death due to early tumor progression. It is suggested that the treatment outcome may be improved by preventing the lymphopenia associated with treatment.

The target definition in radiotherapy for GBM differs among guidelines, and no optimal target definition has been determined. In recent years, it has been reported that there is a correlation between the normal brain volume receiving the moderate dose of 25 Gy or more and the frequency of severe lymphopenia. It has been suggested that a large prophylactic irradiation could induce severe lymphopenia and, as a result, the treatment outcome could be adversely affected.

Based on this background, this report reviews recent findings of the immune response and the immunotherapy for GBM and the novel role of radiotherapy and propose the need to develop an optimal radiotherapy that takes its effect on the immune function into account.

### Overview of immunotherapy for GBM

The GBM has been reported to have similar characteristics to tumors that respond well to immunotherapy, and immunotherapy is expected to be effective in the treatment. For example, a high CD4 + /CD8 + cell count ratio of infiltrating lymphocytes in the tumor was associated with a poor prognosis [1], and GBM with a high mutational burden responded significantly to the administration of ICI nivolumab [2]. In addition, it was reported that ICI improved the survival rate in the murine glioma model [3]. Based on these results, randomized Phase III trials using ICI for recurrent and newly diagnosed GBM and clinical trials of vaccine therapy were conducted.

The CheckMate 143 prospective phase III randomized clinical trial compared bevacizumab with nivolumab in patients with recurrent GBM after standard therapy. The results showed no significant difference in overall survival (OS) between the two therapies, and progression-free survival (PFS) was significantly better in the bevacizumab group. Inhibitors of vascular endothelial growth factor (VEGF), including bevacizumab, are known to cause a phenomenon, the so-called pseudo-response, in which the tumor and edematous region appears to shrink on images due to the effects of inhibiting angiogenesis and reducing the permeability of the blood–brain barrier (BBB). Although the clinical impact of differences in PFS is unclear, it was established that at least nivolumab did not significantly improve treatment outcomes [4].

The CheckMate 498 prospective phase III randomized clinical trial compared the Stupp regimen with temozolomide (TMZ) replacement with nivolumab (NIVO) in newly diagnosed GBM patients with negative methylation of the O^6^-methylguanine-DNA methyltransferase (MGMT) gene promoter region. However, in that study, both OS and PFS were significantly lower in the NIVO group than in the TMZ group. Treatment-related adverse events (Grade 3/4) were 22.0% in the NIVO group and 25.1% in the TMZ group [5].

In the CheckMate 548 prospective phase III randomized clinical trial, 716 newly diagnosed GBM patients with methylated MGMT promoter were randomly assigned at 1:1 to receive either ICI (nivolumab) or a placebo in addition to the conventional Stupp regimen. The result was no significant difference between the two groups in either of OS or PFS, and no efficacy of adding ICI was demonstrated. Treatment-related adverse events (Grade 3/4) were 52.4% in the ICI added group and 33.6% in the placebo group [6].

Based on the above results, the efficacy of PD-1/PD-L1-mediated immunotherapy for GBM has not been established, but some immunotherapies have been shown to be effective. Peptide vaccine (rindopepimut) targeting the epidermal growth factor receptor (EGFR) variant III expressed on GBM cells in 20–30% of patients [7] showed encouraging result in median OS of 24 months in phase II trial. Rindopepimut was evaluated in the multicenter phase III trial (ACT IV), however, it failed to show any increase in OS of patients with newly diagnosed GBM by adding rindopepimut to the standard oral temozolomide (median OS was 20.1 months in the rindopepimut arm and 20.0 months in the control arm, *p* = 0.93) [8]. As other immunotherapies, treatment using patient-derived dendritic cells have been reported [9]. Recently, adding autologous tumor lysate-loaded dendritic cell vaccination to the standard care has significantly improved patient survival in both newly diagnosed and recurrent GBM patients (median OS was 19.3 months in the immunotherapy arm and 16.5 months in the control arm for newly diagnosed GBM (*p* = 0.002), 13.2 and 7.8 months for recurrent GBM (*p* < 0.001)) [10]. Combination immunotherapy is also attracting attention, and a variety of different combination strategies are under investigation [11].

It has been reported that GBM has an intrinsic resistance to immune responses and it also easily acquires resistance to immunity. In a recent review, Jackson et al. categorized the mechanisms of the resistance acquired by GBM to immunotherapy into intrinsic, adaptive, and acquired resistance [12]. They described these as that “intrinsic resistance prevents the initiation of a response; adaptive resistance deactivates tumor-infiltrating immune cells, and acquired resistance protects a tumor from elimination when subject to attack by the immune system”. The GBM has also been reported to suppress systemic immunity. Mechanisms include inhibition of migration of immune cells into the brain through the BBB, sequestration of immune cells into the bone marrow, and inhibition of dendritic cell and T cell responses [12]. By elucidating the mechanism by which GBM evades attack from the immune system, it is expected that the treatment outcomes of GBM improve, as well as that it will also be useful in the treatment of immunotherapy-resistant malignant tumors other than GBM. Therefore, the focus in immune-oncology research is shifting to the development of strategies that target various resistance mechanisms. It should be noted that not only chemotherapy [13] and steroids [14] but irradiation administered for treatment could also attenuate the immune response to the tumor as described in the next section.

### Radiation effects on the immune system

Radiation has been widely reported to affect the immune system and to work against tumors, and many excellent reviews exist [15–18]. Radiotherapy is a double-edged sword that both enhances and attenuates the immune response to the tumors.

Irradiation promotes the release of tumor antigens from tumor cells, increases the number of lymphocytes infiltrating into tumors, and enhances the immune presentation by dendritic cells. In addition, irradiated tumor cells show altered expressions of molecules involved in programmed cell death, such as cell surface FAS ligands and PD-L1, which may enhance the efficacy of ICI [16]. Such immune responses to the tumors are thought to be enhanced by single large-dose stereotactic irradiation when compared with conventional radiotherapy of 1.8–2 Gy per fraction [15, 17].

It has also been suggested that irradiation could increase the proportion of regulatory T cells that suppress the immune responses, and that it may have some effect on myeloid-derived immunosuppressive cells [17]. In addition, naive T cells that circulate in the blood and circulate throughout the body are extremely sensitive to radiation, and it has been reported that the 90% lethal dose is about 3 Gy, and cell death may occur at a dose of about 0.5 Gy [19]. Therefore, lymphocytes circulating in the high-dose area around the tumor as well as in the low-dose area could be destroyed, which would lead to lymphopenia. Yovino et al. calculated the amount of blood to be irradiated according to the number of irradiations, dose rate, and irradiation field size, assuming certain conditions, such as cardiac output, blood flow in the brain, and total blood flow. They reported that the proportion of blood exposed to 0.5 Gy or more increased with a larger number of irradiations, lower dose rates, and larger irradiation fields. According to the assumption, 98.8% of circulating blood had received 0.5 Gy or more during the total treatment of 60 Gy in 30 fractions for GBM with a planning target volume (PTV) diameter of 8 cm [20]. Lambin et al. distinguished high out of field doses, large irradiation volumes, and long irradiation time as risk factors for radiation-induced lymphopenia, as well as doses to immune-related risk organs, and proposed that blood is also an immune-related risk organ [21].

The normal brain, with its abundant blood flow, is considered an immune-related risk organ because of the small volume of bone marrow and lymphoid tissue irradiated during radiotherapy for GBM. Rudra et al. reported a significant increase in the frequency of acute severe lymphopenia (ASL) (i.e., total lymphocyte count < 500 cells/ml within 3 months after radiotherapy) in patients whose brain V25Gy (volume receiving 25 Gy or more) exceeded 40% [22].

### Relationship between lymphopenia and clinical outcomes

The mechanism by which radiation damages tumor cells is thought to be mainly through DNA damage, but early in the history of radiotherapy studies have suggested that the immune system also participates. Stone et al. investigated doses to control fibrosarcoma in mice and found that high doses were required to control tumors in immunosuppressed mice, whereas mice in which the immune response was activated by bacterial infection reported significantly lower doses were required [23]. Grossman et al. reported that patients with a CD4-positive lymphocyte count of less than 200 cells/mm^3^ at 2 months after initiating therapy for high-grade gliomas had a poor prognosis, and that the cause of the poor prognosis was tumor progression rather than infection. They suggested that severe and long-lasting reductions in CD4 lymphocytes could attenuate the therapeutic effect [24]. Recently, Mohan et al. reported that proton therapy significantly reduced the incidence of ASL compared with X-ray intensity-modulated radiotherapy (IMRT) [25]. In addition to brain V20Gy, they identified gender (female) and low pretreatment lymphocyte count as risk factors for lymphopenia, but it was noted females had a significantly better OS. They speculated that the reason for the higher frequency of lymphopenia in females is a sex-based difference in the cerebral blood flow [26] and metabolism [27], and that the reason why females had better OS was the possibility of a higher sensitivity of females to TMZ. However, due to the insufficient number of patients, the cause is not fully explained. Elucidation of this mechanism could lead to improved treatment outcomes. It has been reported that lymphopenia before and after radiotherapy correlates with the prognosis in various tumors other than brain tumors (head and neck squamous cell carcinoma, cervical cancer, esophageal cancer, non-small cell lung cancer, and pancreatic cancer) [28]. It has also been reported that lymphopenia may attenuate the effects of ICI [29].

### Target definitions for GBM radiotherapy planning

The effectiveness of ICI in lung cancer was demonstrated in the PACIFIC trial [30], and the influence of immune responses on tumor control has attracted attention and various studies have been conducted. In a large retrospective study, the group receiving prophylactic nodal irradiation had a significantly worse prognosis than the group receiving radiotherapy to only primary lesions and radiographically involved regional lymph nodes [31]. In addition, as a secondary analysis of the RTOG0617 study, which verified the significance of dose escalation for non-small cell lung cancer, the dose to the blood and the prognosis were examined. It has been suggested that irradiation of immune cells circulating in the blood are important for the tumor control [32], and a recent review reached the same conclusion [33]. Based on these results, it is possible that the antitumor effect could be reduced by performing large prophylactic irradiation, and increase the importance of optimal target definition.

Analysis of GBM recurrence sites by CT imaging and autopsy showed that more than 80% of recurrent lesions occurred within 2–3 cm of the resection cavity [34–37], indicating that tumor cells are most abundant within 2 to 3 cm around the resection cavity and residual tumors. In addition, Kelly et al. and Earnest et al. reported the result of serial biopsies for patients with glial neoplasm and they found tumor cells in the edematous area (i.e., hypo-density area on CT images and high-intensity area on the T2-weighted images) around the tumor [38, 39]. Halperin et al. reported that if radiation portals had been designed to cover the contrast-enhancing volume and peri-tumoral edema with a 3 cm margin, the portals would have covered histologically identified tumors in all cases [36]. Current radiotherapy target definition was based on these reports, however, the number of cases in these reports was small, and some used CT images to determine the range of the edematous area. It cannot be said that this definition is still optimal even with modern diagnostic imaging and radiotherapy techniques. In addition, there is no consensus as to whether the T2 hyper-intense region should be the gross tumor volume (GTV) or it should be the clinical target volume (CTV), and therefore various target definitions have been set according to guidelines and clinical trials. Typical target definitions for each guideline and group are shown in Table 1 [40–46]. For example, in the Radiotherapy and Oncology Group (RTOG), GTV1 was defined as the surgical resection cavity plus residual tumor plus surrounding edema, CTV1 as GTV1 plus a margin of 2 cm, and PTV1 as CTV1 plus a margin of 3–5 mm. After irradiation of 46 Gy in 23 fractions to PTV1, 14 Gy in 7 fractions is added to the resection cavity plus the residual enhancing tumor (GTV2) with the same GTV-CTV and CTV-PTV settings [40]. Differently, in the European Organization for Research and Treatment of Cancer (EORTC), GTV was defined as the surgical resection cavity plus the residual tumor, CTV as GTV plus a margin of 2 cm, and PTV as CTV plus a margin of 3–5 mm. A total irradiation of 60 Gy in 30 fractions is delivered to the PTV without field shrinkage [45]. In addition, at the MD Anderson cancer center (MDACC), after irradiation of 50 Gy in 25 fractions with the same settings as EORTC, the GTV-CTV margin was set to 0 mm, and the PTV set to GTV plus a margin of 3–5 mm and 10 Gy in 5 fractions was added [46]. In the first 50 Gy irradiation, the fluid-attenuated inversion recovery (FLAIR) high-intensity area that is considered to be tumor by radiation oncologist may be included in the CTV.

**Table 1:** Typical target definitions for each guideline and group

	Phase	GTV	CTV*	Dose to PTV**
RTOG [40]	First	Surgical resection cavity + residual tumor (T1E) + surrounding edema	GTV + 20 mm (15–30 mm)	46 Gy in 23 fractions
	Second	Surgical resection cavity + residual tumor (T1E)	GTV + 20 mm (15–30 mm)	14 Gy in 7 fractions
JCOG [41]	First	Surgical resection cavity + residual tumor (T1E) + surrounding edema	GTV + 15 mm	50 Gy in 25 fractions
	Second	Surgical resection cavity + residual tumor (T1E)	GTV + 15 mm	10 Gy in 5 fractions
NCCTG [42]	First	Surgical resection cavity + residual tumor (T1E) + surrounding edema	GTV + 20 mm	50 Gy in 25 fractions
	Second	Surgical resection cavity + residual tumor (T1E)	GTV + 20 mm	10 Gy in 5 fractions
ECOG [43]	First	T1E + surrounding edema on pre-surgery MRI/CT scan	GTV + 20 mm	45 Gy in 25 fractions
	Second	T1E on pre-surgery MRI/CT scan	GTV + 20 mm	14.4 Gy in 8 fractions
ABTC [44]	First	Surgical resection cavity + residual tumor (T1E) + surrounding edema	GTV + 5 mm	46 Gy in 23 fractions
	Second	Surgical resection cavity + residual tumor (T1E)	GTV + 5 mm	14 Gy in 7 fractions
EORTC [45]	Single	Surgical resection cavity + residual tumor (T1E)	GTV + 20 mm (15–30 mm)	60 Gy in 30 fractions
MDACC [46]	First	Surgical resection cavity + residual tumor (T1E)	GTV + 20 mm + FLAIR HIA considered to be tumor	50 Gy in 25 fractions
	Second	Surgical resection cavity + residual tumor (T1E)	GTV	10 Gy in 5 fractions

*GTV* gross tumor volume, *CTV* clinical target volume, *PTV* planning target volume, *T1E* gadolinium enhancing lesion on post-contrast T1-weighted MRI image, *FLAIR* fluid-attenuated inversion recovery, *HIA* high-intensity area, *RTOG* radiation therapy oncology group, *JCOG* Japan clinical oncology group, *NCCTG* north central cancer treatment group, *ECOG* eastern cooperative oncology group, *ABTC*: adult brain tumor consortium, *EORTC*: European organization for research and treatment of cancer, *MDACC* MD Anderson cancer center

*The CTV is modified based on the structures considered to be anatomical barriers, such as bones, falx, and cerebellar tentorium

**The PTV is commonly defined as CTV plus a margin of 3–5 mm

For elderly and/or poor performance status GBM patients, hypo-fractionated regimens of 40 Gy in 15 fractions and 34 Gy in 10 fractions were shown to be comparable to the conventional 60 Gy in 30 fractions regimen in terms of survival in prospective phase III trials [47, 48]. The addition of TMZ to 40 Gy in 15 fractions in elderly patients resulted in longer survival than radiotherapy alone [49]. The target definition and result of these trials are summarized in Table 2.

**Table 2:** Trials evaluating hypo-fractionated radiotherapy for elderly and/or poor performance status GBM patients

Author, year	Trial design	Target definition	Dose fractionation	Number of patient	Concurrent therapy	Median OS	Toxicity
Roa 2004 [47]	Prospective phase III randomized	PTV1: contrast-enhancing tumor plus peri-tumoral edema on the pre-operative CT/MRI scan with a 20 mm margin PTV2: Contrast-enhancing tumor on the pre-operative CT/MRI scan with a 25 mm margin	Conventional RT (CRT) arm: 46 Gy in 23 fractions (PTV1) 14 Gy in 7 fractions (PTV2) Short-course RT (SRT) arm: 40 Gy in 15 fractions (PTV1)	CRT: 47 SRT: 48	None	CRT arm: 5.1 months SRT arm: 5.6 month (*p* = 0.57)	Seventeen (49%) of 35 patients in the CRT arm required a posttreatment increase in total daily dose from the beginning of treatment compared with 10 (23%) of 43 patients in the SRT arm (chi-square test, *p* = 0.02)
Malmström 2012 [48]	Prospective phase III randomized	Not reported	Conventional RT (CRT) arm: 60 Gy in 30 fractions Hypo-fractionated RT (HRT) arm: 34 Gy in 10 fractions	CRT: 100 HRT: 98	None	CRT arm: 6.0 months HRT arm: 7.5 months (p = 0.24)	Reported treatment-related adverse events were mostly mild and were similar for the two radiotherapy groups. Most reported adverse events were related to the underlying disease rather than to treatment
Perry 2017 [49]	Prospective phase III randomized	GTV: contrast-enhancing volume on the postsurgical MRI scan and included the surgical bed CTV: GTV plus a margin of 15 mm respecting anatomical boundaries PTV: CTV plus a margin of 5 mm	40.05 Gy in 15 fractions (PTV)	RT alone: 281 RT + TMZ: 281	With or without TMZ	RT alone: 7.6 months RT + TMZ: 9.3 months (*p* < 0.001)	RT + TMZ was associated with a slightly higher rate of adverse events than RT alone. The rates of grade 3 or 4 events with RT + TMZ versus RT alone were as follows: Lymphopenia, 27.2% versus 10.3% Thrombocytopenia, 11.1% versus 0.4% Neutropenia, 8.3% versus 0.8%

*GTV* gross tumor volume, *CTV* clinical target volume, *PTV* planning target volume, *TMZ* temozolomide, *OS* overall survival, *RT* radiotherapy

Wernicke et al. retrospectively reviewed various target definitions and clinical outcomes during radiotherapy for GBM and reported that there were no clear differences in recurrence patterns or survival rates due to differences in margin size and target settings. Recently, Kumar et al. compared the RTOG and MDACC methods in a randomized phase II trial and reported that the MDACC method group patients had significantly better OS and PFS [50]. In the multivariate analysis, age, extent of resection, and percentage of brain irradiated ≥ 57 Gy were considered predictors of OS and PFS. In this study, the number of cases was small, and there was no information regarding molecular and genetic markers, such as the MGMT promoter methylation status and the presence of iso-citrate dehydrogenase (IDH) mutations. It was not possible to determine whether differences in the target definition alone caused the differences in OS and PFS, but at least it was suggested that a smaller target definition did not result in a worsened treatment outcome, but rather improved the treatment outcome.

### Past results and future perspective

No immunotherapy has been established as effective for GBM at present; however, there is little doubt that one of the reasons for the treatment resistance of GBM is its high ability to evade attacks from the immune system. Novel immunotherapies other than PD-1/PD-L1-mediated tumor immunity, personalized immunotherapy using patient-specific tumor antigens [51], and combinations with multiple immunotherapeutic agents are currently under active investigation [11]. Radiotherapy enhances the immune response to the tumor, while a large irradiation field and too much fractionation could weaken the immune function. Therefore, irradiation to the minimum necessary target and a smaller number of fractions (i.e., hypo-fractionation) could maximally activate the immune function. In addition, it is thought that treatment that can reduce low-dose irradiation around the target, such as particle therapy, would be more effective.

A number of phase I and phase II trials of hypo-fractionated radiotherapy for non-elderly/poor performance status GBM patients have been reported [52–55] (Table 3). Studies using high biological doses have reported a reduced frequency of central field recurrences [53, 54], suggesting that high-dose hypo-fractionation may improve central tumor control. All reported favorable results with a median OS of about 20 months, but a recent systematic review showed no significant improvement in OS with hypo-fractionation, partly because of the variety of target definitions and dose fractionations [56]. Here it must be borne in mind that the risk of brain necrosis increases with increasing biological doses [53, 54], and there is also a report that hypo-fractionated radiotherapy is associated with increased brain necrosis [57]. There is a need for more precise targeting and minimizing the brain volume irradiated to safely perform high-dose hypo-fractionated radiotherapy.

**Table 3:** Trials evaluating hypo-fractionated radiotherapy for non-elderly/poor performance status GBM patients

Author, year	Trial design	Target definition	Dose fractionation	Number of patient	Concurrent therapy	Median OS	Toxicity
Chen 2011 [52]	Prospective phase I	GTV: Contrast-enhancing residual tumor on the T1-weighted pre-RT brain MRI scan plus the entire surgical cavity CTV: T2-weighted abnormality on T2-weighted brain MRI PTV1: GTV plus a margin of 5 mm PTV2: CTV plus a margin of 5 mm	60 Gy (PTV1), 45 Gy (PTV2) in 20 fractions (Level 1) 60 Gy (PTV1), 40.5 Gy (PTV2) in 15 fractions (Level 2) 60 Gy (PTV1), 36 Gy (PTV2) in 12 fractions (Level 3) 60 Gy (PTV1), 30 Gy (PTV2) in 10 fractions (Level 4)	Level 1: 3 Level 2: 3 Level 3: 4 Level 4: 6	TMZ	16.2 months	Three patients underwent a second craniotomy and were diagnosed with extensive brain necrosis (1 at Level 1, 1 at Level 2, and 1 at Level 4) One patient who was treated at dose Level 2 developed Grade 4 vision loss 7 months after RT
Tsien 2012 [53]	Prospective single institutional	GTV: residual gross tumor or resection cavity, based on the contrast-enhancing T1-weighted MRI CTV: GTV plus a margin of 15 mm (within the skull) PTV1: CTV plus a margin of 5 mm PTV2: GTV plus a margin of 5 mm	60 Gy (PTV1), 66 Gy (PTV2) in 30 fractions (Level 1) 60 Gy (PTV1), 72 Gy (PTV2) in 30 fractions (Level 2) 60 Gy (PTV1), 75 Gy (PTV2) in 30 fractions (Level 3) 60 Gy (PTV1), 78 Gy (PTV2) in 30 fractions (Level 4) 60 Gy (PTV1), 81 Gy (PTV2) in 30 fractions (Level 5)	Level 1: 1 Level 2: 12 Level 3: 9 Level 4: 7 Level 5: 9	TMZ	20.1 months	The number of dose-limiting toxicities (DLT) with estimated probabilities was: 0 of 1 patient at 66 Gy (2%), 0 of 12 at 72 Gy (8%), 2 of 9 at 75 Gy (14%), 3 of 7 at 78 Gy (23%), 2 of 9 at 81 Gy (36%) * DLT: any grade III or IV irreversible CNS toxicity, nonhematologic, non-CNS grade IV toxicity, or any grade V toxicity
Iuchi 2014 [54]	Prospective phase II	GTV: contrast-enhancing residual tumor plus the entire surgical cavity on MRI PTV1: GTV plus a margin of 5 mm PTV2: PTV1 plus a margin of 15 mm PTV3: High-intensity region on FLAIR images without margins	68 Gy (PTV1) 40 Gy (PTV2) 32 Gy (PTV3) in 8 fractions over 10 days	46	TMZ	20.0 months	The incidence of radiation necrosis was 10.9% surrounding the original tumor, 21.7% in the sub-ventricular zone, and 10.9% in both
Mallick 2018 [55]	Prospective phase II randomized	CTV50: enhancing tumor and edema as seen in the pre-operative T2-weighted/FLAIR MRI with a 15–20 mm margin all around CTV 60: T1 contrast-enhancing tumor with a 15 mm margin PTV50: CTV50 plus a margin of 3 mm PTV60: CTV60 plus a margin of 3 mm	Conventional RT (CRT) arm: 60 Gy in 30 fractions (PTV60) 50 Gy in 25 fractions (PTV50) Hypo-fractionated accelerated RT (HART) arm: 60 Gy (PTV60) 50 Gy (PTV50) in 20 fractions	CRT: 38 HART: 45	TMZ	CRT arm: 18.1 months HART arm: 25.2 months (p = 0.3)	Only one patient of HART arm had documented radio necrosis

*GTV* gross tumor volume, *CTV* clinical target volume, *PTV* planning target volume, *FLAIR* fluid-attenuated inversion recovery, *TMZ* temozolomide, *OS* overall survival, *RT* radiotherapy

As shown in Table 1, the ABTC trial had the smallest margins among the previous trials, but a retrospective recurrence pattern analysis of patients who were treated with ABTC margin definitions showed 80% in-field recurrence and 6% marginal recurrence, rates that were similar to wide margin trials [44]. In addition, Paulsson et al. compared the recurrence patterns, OS, and PFS of patients with different CTV margins between 5 and 20 mm, and found no significant differences in recurrence patterns, OS, and PFS due to differences in the margin sizes [58]. These results suggest that there is little need to provide a uniform wide margin of about 2 cm.

Tsien et al. reported that patients whose treatment did not include the region of increased ^11^C-methionine-positron emission tomography (MET-PET) uptake showed an increased risk of non-central failure [53]. In addition, Miwa et al. performed hypo-fractionated IMRT with MET-PET data for target delineation, and reported favorable results with a median OS of 20.0 months [59]. In addition to MET-PET, trials of personalized target definitions using diffusion tensor images and deep learning were recently reported [60]. Although the efficiency of these techniques on clinical results has not been clearly established, the target definition using them could be more reasonable than adding uniformly wide margins. From the viewpoint of considering blood as an organ at risk, investigation of target settings using the information of cerebral blood flow that can be acquired by MRI is also promising. High-dose hypo-fractionated radiotherapy with a minimal target definition using high-precision imaging, and combined use of single or multiple immunotherapies could be expected to improve treatment outcomes.

## Conclusions

Due to the present knowledge about the efficacy of immunotherapy for tumors and the immune function against tumors, the role of radiotherapy is changing significantly from killing tumor cells locally to activating immune functions. At the same time, immune cells in the blood have been newly recognized as risk organs for radiotherapy. It is necessary to develop optimal radiotherapy methods considering the new roles of radiotherapy and risk organs.
